# Hierachical epicuticular wax coverage on leaves of *Deschampsia antarctica* as a possible adaptation to severe environmental conditions

**DOI:** 10.3762/bjnano.13.71

**Published:** 2022-08-22

**Authors:** Elena V Gorb, Iryna A Kozeretska, Stanislav N Gorb

**Affiliations:** 1 Department of Functional Morphology and Biomechanics, Zoological Institute, Kiel University, Am Botanischen Garten 9, 24118 Kiel, Germany; 2 National Antarctic Scientific Center of Ukraine, Taras Shevchenko Boulevard 16, 01601 Kyiv, Ukraine

**Keywords:** cryo-SEM, microstructure, plant, surface, wax projection

## Abstract

Using cryo scanning electron microscopy, the surface micromorphology of vegetative (leaf blade and ligule) and generative (pedicel and outer glume) organs in *Deschampsia antarctica*, one of the only two flowering plants native to Antarctica, was examined. Whereas the pedicel and outer glume were wax-free, both leaf sides had a prominent epicuticular wax coverage consisting of two superimposed layers: polygonal rodlets formed by fused irregular platelets (the lower wax layer) and membraneous platelets (the upper wax layer). Although the adaxial (inner) and abaxial (outer) leaf surfaces showed a similar microstructure of the wax coverage, they differed in the thickness ratio between lower and upper wax layer. The ligule bore a very loose wax coverage composed of separate scale-like projections or clusters of them. We suppose that the two-layered wax densely covering both leaf surfaces might contribute to the plant adaptation to severe environmental conditions in Antarctica due to an increase of its resistance against cold temperatures, icing, harmful UV radiation, and dehydration. The presence of the epicuticular wax on the abaxial leaf side and the ligule as well as the hierarchical structure of the wax coverage on both leaf surfaces is described in *D. antarctica* for the first time.

## Introduction

The Antarctic hair grass *Deschampsia antarctica* É. Desv. (Poaceae) is one of the only two flowering plants native to Antarctica, where it inhabits northern and western parts of the Antarctic Peninsula and adjacent islands free of permanent ice and snow in summer [1]. These perennial, 3–20 cm high plants have leaves with narrow folded or V-shaped ribbed leaf blades and true panicle inflorescences. They grow either as a separate clump or form a dense and continuous cover spreading from one to several hundred square meters and occupy all favourable places for growing, such as rocks, hollows, corniches, and beaches, either poor or overrich with semidecomposed organics soils [2–3].

Compared to the other flowering plant species native to Antarctica, the Antarctic pearlworts *Colobanthus quitensis* Kunth Bartl. (Caryophyllaceae), which is rather rare and mainly relays on an avoidance strategy (i.e., it grows near higher clumps of *D. antarctica* or in hollows between stones avoiding direct influence of unfavorable abiotic factors), the Antarctic hair grass not only uses the avoidance strategy, but also grows in separate agglomerations or forms thick covers on the unprotected ground surface [3]. Especially in the latter case, *D. antarctica* plants are fully exposed to the cold desert climate of Antarctica, including low temperatures, moisture deficit, and strong ultraviolet radiation [2,4].

In view of the remarkable success of this plant species in the colonization of the Maritime Antarctica (i.e., ice- and snow-free lands along the Antarctic Peninsula, its associated islands, and coastal regions around the rest of the Antarctic continent accounting for less than 1% of the Antarctica territory [5]), intensive experimental investigations were recently performed on structural, physiological, genetical, and molecular biological characteristics of *D. antarctica*, in order to find specialized adaptations to severe environmental conditions that ensure this evolutionary success [2–4,6]. Structural studies that have been focused mainly on anatomical and ultrastructural characterization of leaves [4,7–12] did not reveal any qualitative traits allowing for a distinction of this plant from other polar species and explaining its better survival in the Antarctica region. The leaves exhibit xerophytic (i.e., typical for plants growing in arid places) morphological and anatomical attributes, and the general anatomical features of mesophyll cells are similar to those of other plant species growing in cold regions [13] and to those in other grass leaves. However, the surfaces of *D. antarctica* that are exposed to the environment in the first place have not been especially examined in this respect.

In this study, we examined the microstructure of epidermal surfaces in different organs of the *D. antarctica* plant, which are usually exposed to the environment, using cryo scanning electron microscopy (cryo-SEM) allowing for a high-resolution imaging of frozen and fractured samples in native condition, that is, without treatment in strong solvents, such as ethanol or acetone, usually needed in the sample preparation for conventional scanning electron microscopy. This method provided qualitatively new information about the superficial layers on vegetative organs studied in this plant species*.* Based on the obtained results and literature data, we discuss a possible role of the complex, hierarchical epicuticular wax coverage on the leaf surfaces in the adaptation of *D. antarctica* to the severe Antarctic environment. Additionally, the results obtained from this highly specialized plant species might be potentially interesting for biomimetics of technical surfaces or surface coatings exposed to similar harsh environments (e.g., strong UV irradiation, low temperatures, and icing).

## Experimental

### Plant material

*D. antarctica* plants used in this study were collected in the vicinity of Henryk Arctowski Polish Antarctic Station, King George Island, Maritime Antarctica (62°09′50″ S, 58°28′7″ W) during X Ukrainian and XXX Polish Antarctic Expeditions in 2005/2006 (permission АР no. 013-06 to Iryna A. Kozeretska for collection of plant material in the King George Island region, Maritime Antarctica). A separate, small clump of mature plants bearing fully developed flowers was completely dug up including roots, planted into a small plastic flower pot filled with topical soil, and transported to the laboratory.

**Figure 1:**
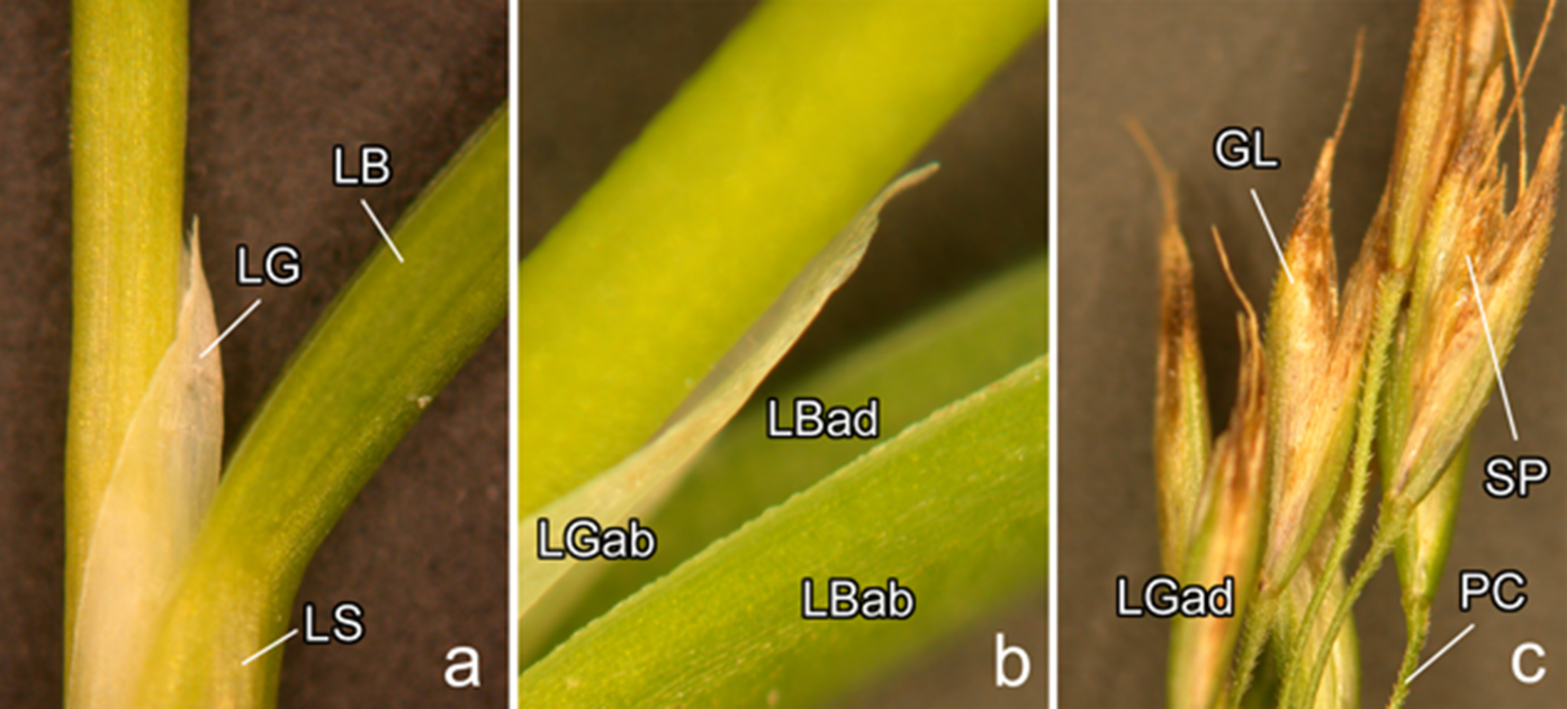
Plant organs and surfaces examined. (a, b) Vegetative organs: the inner, adaxial (LBad) and outer, abaxial (LBab) sides of the leaf blade (LB) and the outer, abaxial (LGab) side of the ligule (LG). (c) Generative organs: the pedicel (PC) and the outer, abaxial side (GLab) of the outer, lower glume (GL)*.* LS, leaf sheath; SP, spikelet.

The surfaces of the following organs were examined (Figure 1): (i) vegetative organs, namely the inner (adaxial) and outer (abaxial) sides of the leaf blade in mature and young leaves as well as the abaxial side of the ligule (a membrane on the adaxial side of the junction of the leaf sheath and leaf blade); and (ii) generative organs, namely the pedicel (a stalk of a single spikelet (collection of flowers (florets) in a grass inflorescence)) and the abaxial surface of the outer, lower glume (a bract below a spikelet). For each organ, we studied three samples belonging to three different plants.

### Microscopy

The leaf and inflorescence surfaces were at first studied on fresh, untreated samples in a binocular microscope Leica MZ 12.5 with a built-in videocamera Leica IC A (Leica Microsystems GmbH, Wetzlar, Germany). For cryo-SEM, we used either entire structures (glume and ligule) or small pieces (ca. 1 cm long for pedicle and ca. 1 cm^2^ for other samples) cut out with a razor blade from the plant. All surfaces were examined in the native state. Additionally, the leaf blade surfaces were studied after treatment in cold chloroform for 20 s. Plant samples were either glued with Tissue-Tek^®^ O.C.T.^TM^ Compound (Sakura^®^ Finetek Europe B.V., Zoeterwoude, Netherlands) to holders or mechanically gripped in a small vice on holders and then frozen in a cryo-stage preparation chamber at −140 °C (Gatan ALTO 2500 cryo-preparation system, Gatan Inc., Abingdon, UK). Frozen samples, either entire or fractured with a cold metal fracture knife in the cryo-stage preparation chamber, were sputter-coated with gold–palladium (6 nm thickness) and examined in frozen condition (−120 °C) in a cryo-SEM Hitachi S-4800 (Hitachi High-Technologies Corporation, Tokyo, Japan) at 3 kV accelerating voltage.

Description of trichomes was performed according to [14]. Types of epicuticular wax structures were identified according to [15]. Morphometric variables of surface features were measured from digital images using the software SigmaScan Pro version 5.0.0 (SPSS Inc., Chicago, USA). The data are given in the text as mean ± SD for *n* = 10.

## Results and Discussion

### Micromorphology of plant surfaces

**Figure 2:**
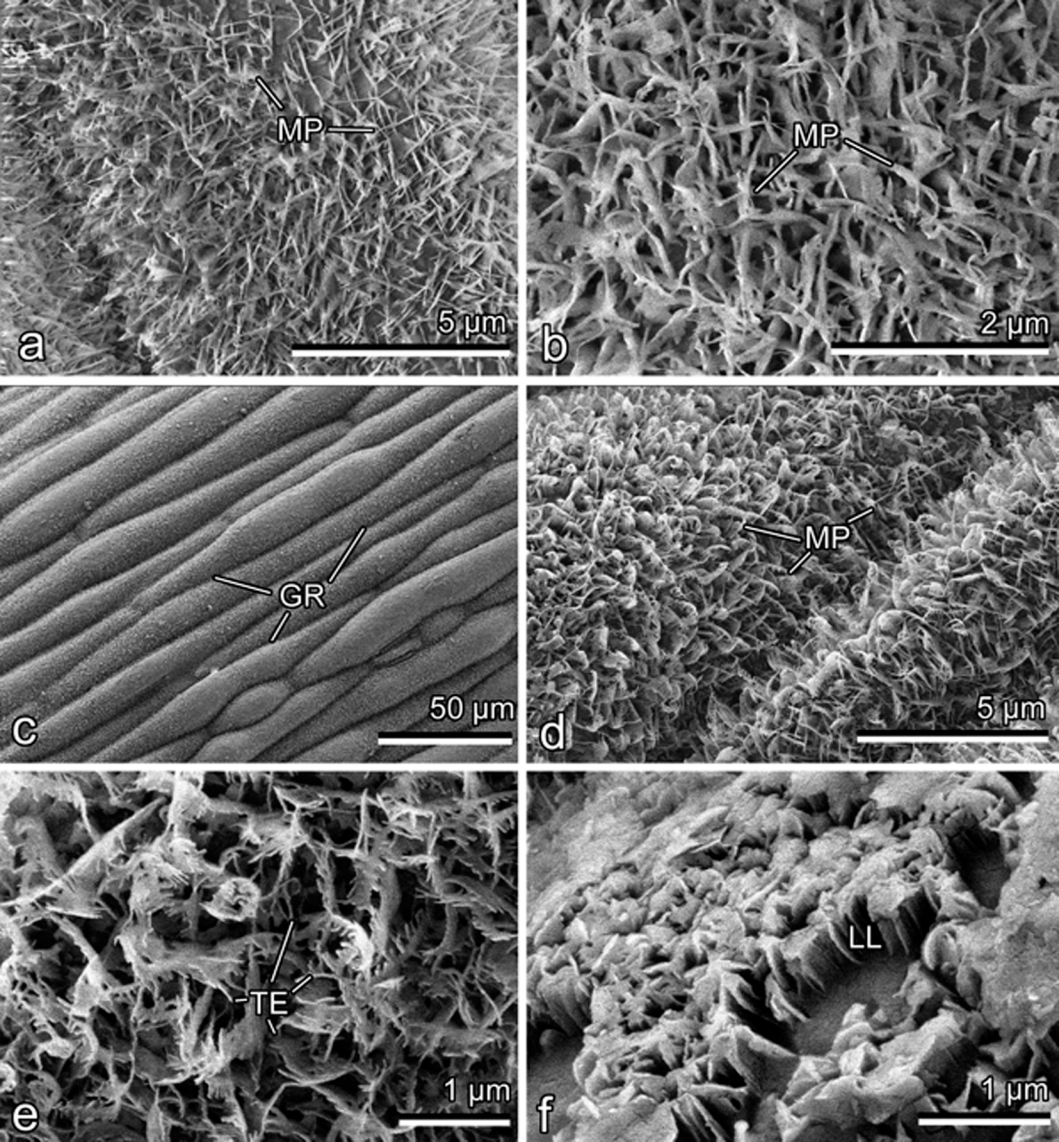
Cryo-SEM micrographs of leaf lamina surfaces. (a, b) Adaxial side. (c–f) Abaxial side. GR, groove; LL, lower wax layer; TE, thread-like extension of the platelet margin; MP, membraneous platelet.

In *D. antarctica*, both leaf lamina sides have characteristic furrowed surfaces, with rather thin and deep grooves, that are caused by shapes of long (>200 μm) and narrow (width: 10.88 ± 1.98 μm) epidermal cells (Figure 2c). In the surface view (i.e., view from above), leaves bear a very dense epicuticular wax coverage composed of numerous membraneous platelets (Figure 2a,d) called here the upper (outer) wax layer. At very high magnifications (probably due to damage by the electron beam), the platelets often looked like being interconnected (Figure 2b). These microscopic, very thin flat projections (length: 0.75 ± 0.08 μm; thickness: 38 ± 6 nm) oriented almost perpendicularly to the epidermal surfaces have no specific arrangement. On the abaxial side, fine thread-like extensions on the margins of the platelets are better seen and the projections seem to be slightly inclined relative to the underlying surface (Figure 2e); these peculiarities are even more expressed in young leaves. On some micrographs, when platelets were removed/damaged, another layer of projections called here the lower (inner) wax layer became exposed (Figure 2f).

**Figure 3:**
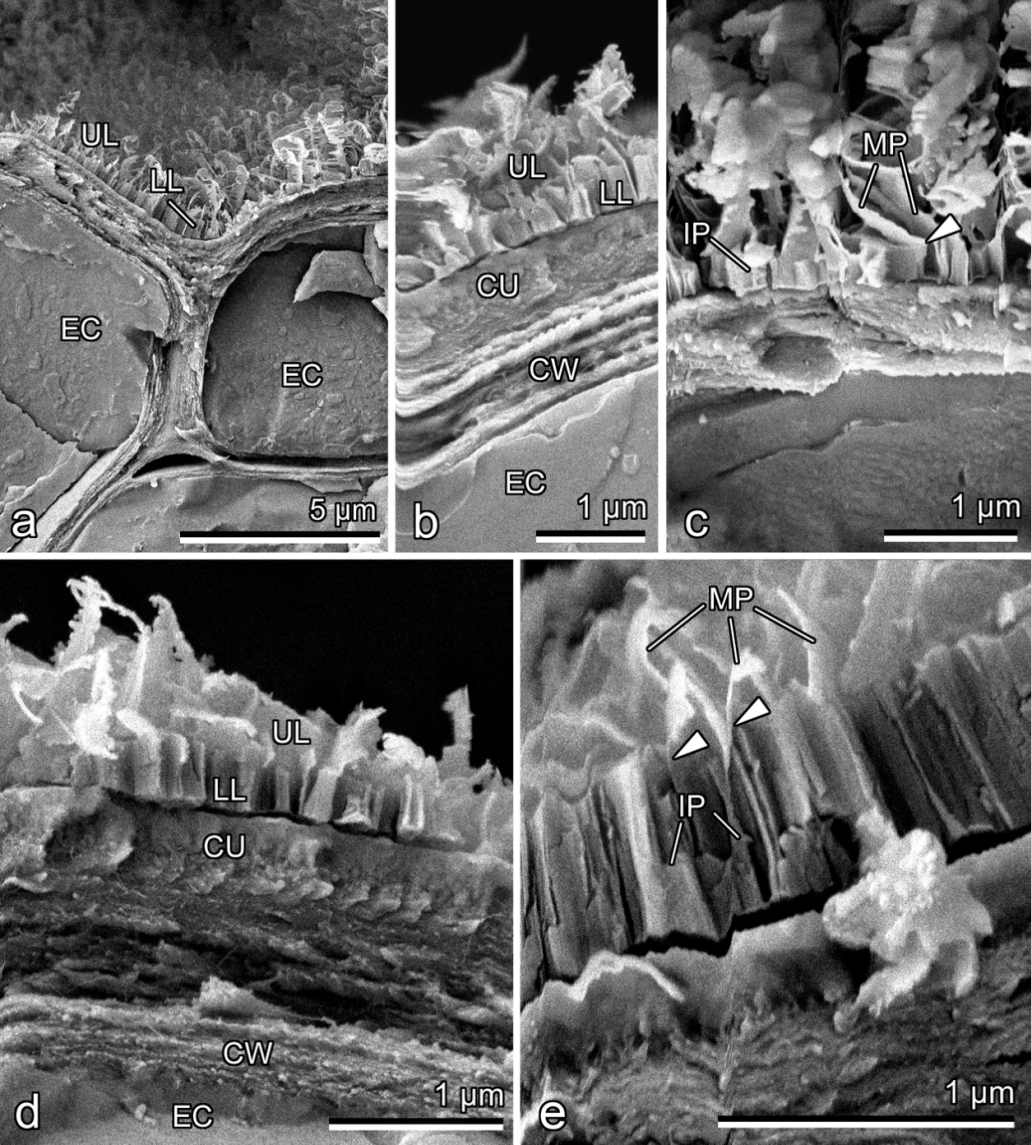
Cryo-SEM micrographs of the fractured epidermis of the leaf lamina. (a–c) Adaxial side. (d, e) Abaxial side. CU, cuticle; CW, outer cell wall of the epidermal cell; EC, epidermal cell; IP, lower irregular platelet; LL, lower wax layer; MP, upper membraneous platelet; UL, upper wax layer. Arrowhead points on the connection between lower and upper platelets.

The fractured samples clearly show the hierarchical organization of the wax coverage on both leaf lamina sides, which is composed of the two superimposed layers of wax projections (Figure 3a,b,d). Whereas the lower layer is rather compact and consists of densely packed polygonal rodlets (length: 0.42 ± 0.10 μm; width: 0.19 ± 0.03 μm) situated perpendicular to the leaf blade surface and formed by tightly placed and fused thin (thickness: 27 ± 4 nm) irregular platelets, the loose upper layer is represented by separate, but entangled, membraneous platelets having elongated irregular shapes (Figure 3b–e). The connection between layers is provided by outgrowths of some lower platelets, which finally turn into upper platelets (Figure 3c,e). The thickness ratio between the lower wax layer and the upper one on the adaxial leaf side is smaller compared to that on the abaxial side (ca. 0.5 vs ca. 0.8, respectively). After treatment with cold chloroform, the epicuticular wax coverage completely disappeared from both leaf lamina surfaces.

A very similar structure of the epicuticular wax coverage (two-layered, with superimposed wax platelets connected through stalks formed as outgrowths of lower projections) was previously found in trapping organs (pitchers) of several representatives of the carnivorous plants from the genus *Nepenthes* (Nepenthaceae) such as *N. alata* Blanco, *N. fusca* Danser, *N. macrophylla* Jebb and Cheek, *N. mirabilis* (Lour.) Druce, and *N. rafflesiana* Jack [16–17]. The waxy (slippery) zone located inside the pitchers is highly specialized for trapping and retaining of insect prey mainly due to contamination of attachment organs of insects [16], reduction of the real contact area between the plant surface and insect adhesive devices [16,18], and absorption of the insect adhesive fluid [19]. Whereas the upper wax platelets are rather fragile and can be easily broken into small pieces and removed from the slippery zone thus contaminating insect attachment organs, the pitchers still remain fully functional in terms of insect trapping owing to the presence of rather stable lower-layer wax projections [16–17]. Taking into account the above consideration about the *Nepenthes* wax, we suggest for leaves of *D. antarctica* that the presence of the two wax layers increases the chance of the wax coverage to fulfill its functions in case when the upper wax layer is for some reasons damaged or removed. Partial damage and/or even removal of the upper layer are very much possible under natural conditions, since the connection of upper projections with the lower layer is realized through very thin and delicate structures (outgrowths of some lower platelets; see Figure 3e), which can readily brake. In this study, we observed such defects in the wax coverage in a few leaf samples, where upper wax platelets were absent and only the lower wax layer remained exposed (see Figure 2f).

**Figure 4:**
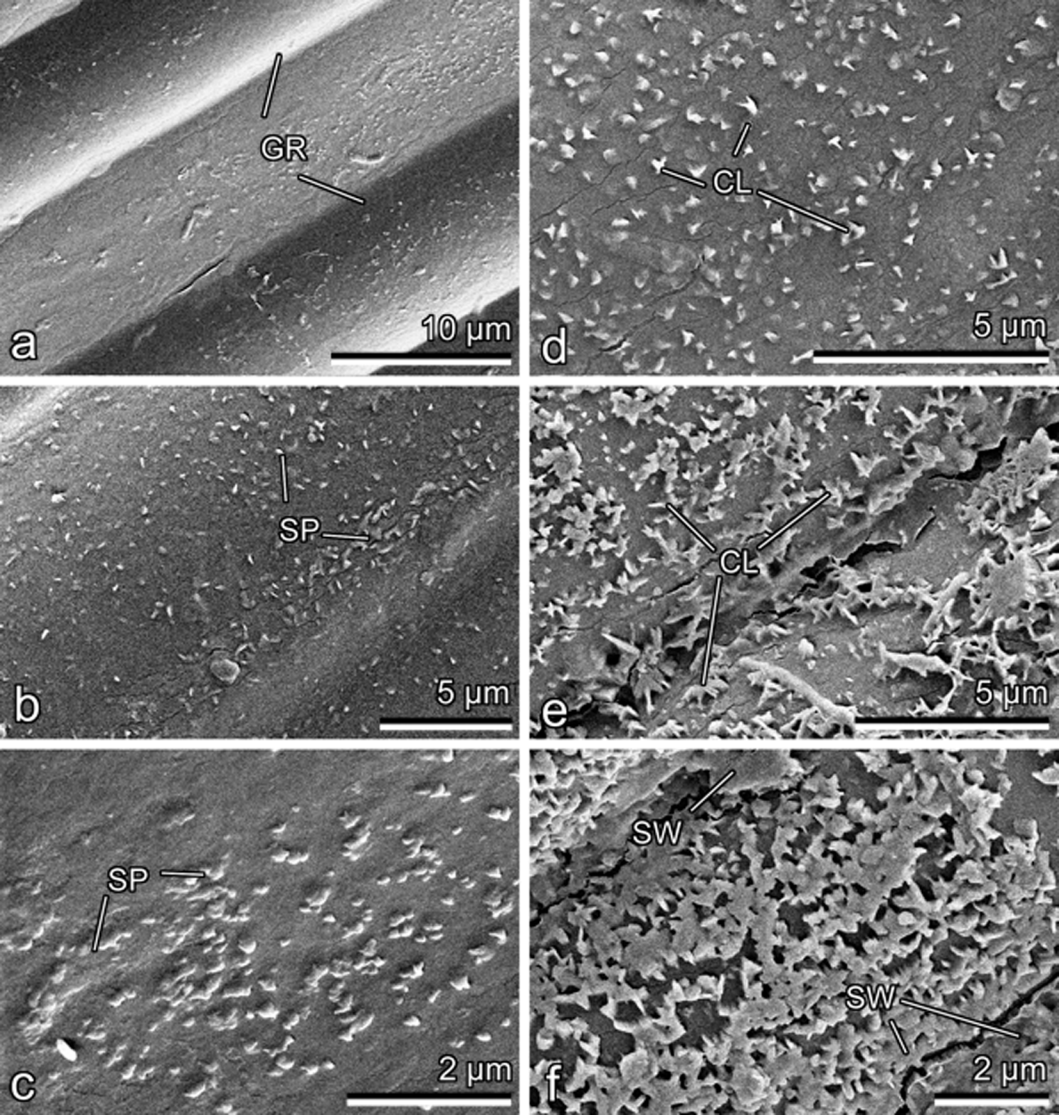
Cryo-SEM micrographs of the abaxial side of the ligule. (a) General view of the surface. (b–e) Wax projections. (f) The surface with smeared wax. GR, groove; CL, cluster of projections; SP, scale-like projection; SW, smeared wax layer.

The coarsely furrowed abaxial surface of the ligule (Figure 4a) bears a very loose epicuticular wax coverage consisting of either (i) single, separate scale-like projections of irregular shapes (however, often almost semicircular) with regular margins, which vary greatly in size and protrude perpendicularly from the pitcher surface (Figure 4b,c), or (ii) larger projections, gradually changing into platelets and arranged in more or less radial clusters (Figure 4d). In case the clusters become interconnected, they build a loose foam-like coverage on the surface (Figure 4e), which turns into a continuous smooth wax layer when mechanically smeared (Figure 4f). The above two types of wax projections very much resemble wax structures observed previously in developing pitchers of the carnivorous plant *N. alata* at the initial developmental stages of wax projections (stages 1 and 2, respectively) [20].

**Figure 5:**
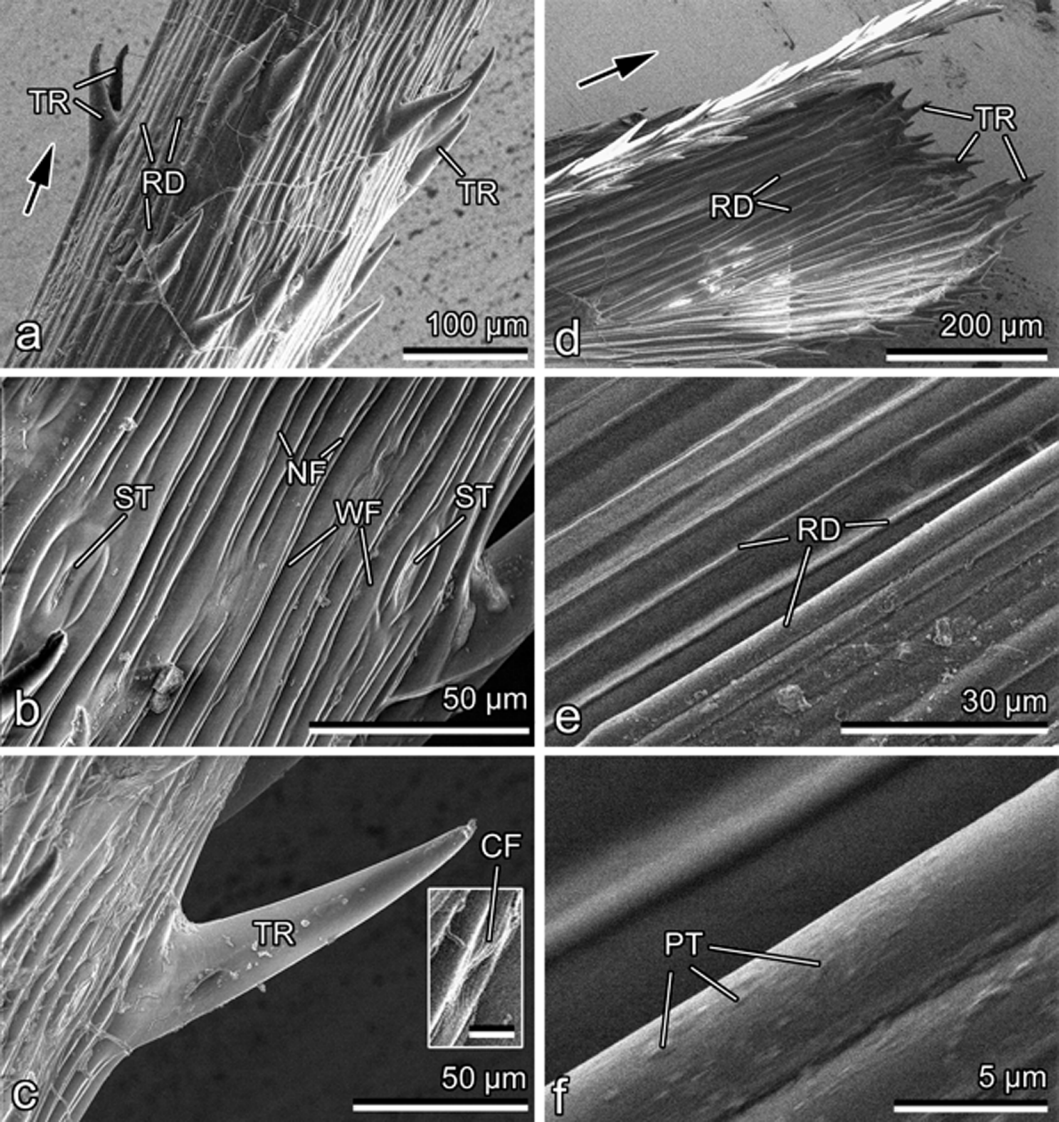
Cryo-SEM micrographs of the surfaces in the generative organs. (a–c) The pedicel. (d–f) The abaxial side of the glume. The inset in (c) shows cuticular folds. CF, cuticular fold; NF, narrow furow; PT, protrusion; RD, ridge; ST, stoma; TR, trichome; WF, wide furrow. The arrows in (a) and (b) show the apical direction for (a–c) and (d–f), respectively. Scale bar in (c): 5 μm.

Both surfaces of the studied generative organs of *D. antarctica* have a noticeably ridged appearance at rather low magnifications (Figure 5a,d). While in the outer glume, the furrows are relatively similar in width (4.52 ± 1.09 μm) (Figure 5e), the pedicel surface shows a clear pattern of alternating narrow (1.38 ± 0.36 μm) and wide (4.66 ± 0.70 μm) furrows (Figure 5b). Additionally, the pedicel is equipped with regularly distributed, inclined, cone-shaped trichomes (length: 99.91 ± 14.00 μm; diameter at the base: 30.48 ± 4.90 μm) with a tapered tip pointing to the apical direction (Figure 5a,c), whereas the glume has smaller trichomes (length: 62.87 ± 7.38 μm; diameter at the base: 13.36 ± 2.51 μm) of the similar type only on margins (Figure 5d). The pedicel bears also stomata scattered over the ridged surface (Figure 5b). At high magnifications, groups of rather flat cuticular folds can be found at some sites on the pedicel surface (Figure 5c, inset), whereas the glume surface shows regularly distributed, rounded/oval, relatively flat, sub-microscopic (length/diameter: 0.54 ± 0.14 μm) protrusions (Figure 5f). Both the pedicel and glume surfaces are lacking prominent epicuticular wax projections.

For the plant species studied, the epicuticular wax coverage has been previously shown (using SEM) and described only on one (adaxial) leaf lamina side [9], where small strands prostrate on each other forming a dense tangle on the entire leaf surface including stomata, whereas the abaxial side lacks the wax coverage. A dense wax coverage on the adaxial leaf surface was later alluded to by Kyryachenko et al. [6] based on the work of Romero and co-workers [10]. However, the latter authors [10] have not referred to the wax, which is nevertheless seen on the SEM images. Also in a more recent review on origin and adaptations of Antarctic flowering plants, Parnikoza et al. [2] mentioned a thick layer of wax covering leaves of *D. antarctica*, however, without any corresponding original literature citation. In another *Deschampsia* species (*D. caespitosa*), the epicuticular wax was reported only on the adaxial leaf side [21]. Using cryo-SEM, we detected and describe here in detail the epicuticular wax coverage on both leaf lamina sides and on the ligule in *D. antarctica* for the first time. Whereas plates [22] and platelets [23–25] as typical wax projections have been previously reported for many Poaceae species, the hierarchical, two-layered epicuticular wax coverage described here in *D. antarctica* is the first to be discovered in grasses.

### Epicuticular wax coverage as an adaptation to severe environmental conditions

The Antarctic hair grass *D. antarctica* is a cushion-forming plant growing along the other vegetation of the Antarctic coast, which consists mainly of about 130 cryptogams (mosses, liverworths, and lichens) and only one other flowering plant species, the Antarctic pearlwort *C. quitensis*, below latitudes of 60°S down to approximately 68°S in the Antarctica [26]. These plants can tolerate the extreme conditions of the Antarctica. They are capable of surviving long winter periods of total or almost total darkness, during which photosynthesis cannot take place, and of growing in short summer times lasting from only a few days to a few months, depending on various factors [27–28].

Growing in the cold desert climate of Antarctica, *D. antarctica* experiences above all effects of very cold and dry conditions. In the Maritime Antarctica, maximum daily air temperatures in summer (January) range between 0 and 6 °C, with mean temperatures of 2.8–6 °C [27,29]. The winter temperatures are between −10 and −20 °C [30], although the Antarctic Peninsula has warmed at a rate of 3.8 °C per century [31]. The mean annual temperature rose by ca. 2.6 °C and the mean summer temperature by ca. 1.5 °C over the past 50 years [32–33]. It is astonishing that this plant is still able to function (both photosynthesis and respiration) at the freezing point, when the rate at which it photosynthesizes drops to ca. 30–40% of that reached during the most favourable conditions estimated in controlled laboratory experiments as 13 °C [27]. Compared to *C. quitensis,* the general cold endurance for *D. antarctica* is very high: The median lethal dose LD_50_ (a measure of the lethal dose of, e.g., a toxin, radiation, or pathogen required to kill half the members of a tested population after a specified test duration) is around −26 °C for *D. antarctica*, compared to −5 °C for *C. quitensis* [3,34]. This remarkable frost resistance of *D. antarctica*, which is achieved through different biochemical adaptations, such as the production of antifreeze proteins that are secreted into the apoplast [35], can be a good explanation why this plant species not only uses the avoidance strategy as *C. quitensis* does, but also grows in separate clumps and can form rather big and thick cushion-like clusters in the Maritime Antarctica. To our opinion, the relatively thick (up to 1.3 μm) two-layered epicuticular wax coverage found in the present study on both leaf lamina sides in *D. antarctica* might also contribute to the plant resistance against low temperatures. The relatively compact lower (inner) wax layer separates well the epidermal cell surface from the cold environment, whereas the upper (outer) wax layer with air-filled spaces between wax projections represents an effective isolation material due to its conspicuous foam-like structure.

Additionally, a recent cryo-SEM experimental study performed with six plant species (*Prunus laurocerasus* L. (Rosaceae) and *Ficaria verna* Huds. (Ranunculaceae) with smooth leaves, *Bellis perennis* L. (Asteraceae) and *Cerastium brachypetalum* Pers. (Caryophyllaceae) with hairy leaves, and *Narcissus pseudonarcissus* L. (Amaryllidaceae) and *Tulipa gesneriana* L. (Liliaceae) with waxy leaves), which in nature may be potentially exposed to freezing temperatures, showed that surface icing and, consequently, the plant cell freezing rate might be significantly reduced due to the presence of three-dimensional epicuticular wax structures [36]. It was found that on waxy leaves, icing started from nucleation sites on the surface of wax projections or in nanoscale depressions between them. This is why the real contact area of ice crystals was limited to a few sites at the nanoscale on or between wax projections. After thawing, water drops flow off the surface and therefore cannot be potentially re-frozen. The study demonstrated the following effects caused by the wax coverage: (i) Air pockets between wax projections prevent direct contact between the plant cuticle and ice crystals, and (ii) after thawing, the fluid water is removed and further re-freezing on the plant surface is averted. Based on the above data, we suppose that the microstructured wax covering both leaf surfaces in *D. antarctica* also could be involved in the anti-icing mechanism of this plant species thus contributing to its frost resistance.

In addition, during the growing season between December and February, the day length is about 20 h in the Antarctic Peninsula [26] and the maximum photosynthetic photon flux density under the full midday sun in January can reach up to 2000 μmol·m^−2^·s^−1^ in the Maritime Antarctica [27,37]. Protection against harmful ultraviolet radiation has been previously reported as one of the principal functions of plant surfaces bearing microstructured epicuticular waxes. It is achieved through the increased reflection and scattering of the incoming radiation by minute wax projections [38–39]. Being covered by the prominent upper (outer) epicuticular wax layer composed of separate/interconnected wax projections of submicrometer size (ca. 0.8 μm long and ca. 40 nm thick), which are almost perpendicularly oriented to the epidermal surface, the distinctly furrowed leaf lamina surfaces of *D. antarctica* seem to be well equipped for the successful scattering of strong radiation. Experimental studies with altered levels of UV-B radiation and *D. antarctica* showed no significant effect of enhanced or reduced radiation on the relative growth rate and leaf photosynthesis of the plants [40].

Moisture, being one of the most important abiotic factors affecting the vegetation in Antarctica, is provided mainly by atmospheric water vapor and local melt supplies from fallen snow, drift snow, and permafrost, whereas stream runoff is extremely rare [28]. Precipitation in the Maritime Antarctica ranges from 100 mm per year (Margarita Bay) to 400 mm per year (South Shetland Islands) [26]. In general, a continuous epicuticular wax layer is known to serve as an transport barrier limiting the uncontrolled water loss in plants [38,41]. The presence of a thick wax coverage has been considered as one of typical xeromorphic features (i.e., structural characteristics that leaves of plants growing under arid conditions have in common in order to drastically lessen the water loss from leaves and are, therefore, classed as adaptational mechanisms to withstand drought [42]). Such leaf characteristics as small epidermal cells, high cell density per area, considerable leaf thickness, thick-walled tissues, thick cuticles, and some other structural features typical of xerophytes were repeatedly revealed in *D. antarctica* by different authors [4,8–12]. As for the epicuticular wax in this plant species, it has been previously reported that the wax on both epidermis and stomata contributes to the resistance of water vapor diffusion from the mesophyll to the outside and to the control of cuticle transpiration, reducing in this way the water loss by the leaf blade [9]. Also, authors associated the epicuticular wax on leaves along with certain anatomical features of the leaf mesophyll cells [4,8] to the protection of *D. antarctica* against dehydration. Based on our cryo-SEM results, we assume that, in this plant species, particularly the compact lower wax layer, which resembles the wax crusts characteristic for many succulents (e.g., Asclepiadaceae and Cactaceae) [15], plays a crucial role in the protection from water loss.

### Biomimetic potential

Since the discovery of the lotus effect [43], different properties of superhydrophobic surfaces in plants, which are highly relevant for modern technologies, such as self-cleaning, fluid drag reduction, or holding air layers at a surface, have been described and their biomimetic applications have been discussed. Phylogenetic trees indicate that superhydrophobicity evolved as a consequence of the conquest of land about 450 million years ago and may be a key innovation in the evolution of terrestrial life [44]. Recently, interesting anti-icing and controlled-icing properties of plant surfaces bearing wax projections and trichome coverage were experimentally studied [36]. The presence of ice on many technical surfaces can potentially cause failure of materials and structures. Therefore, numerous studies were focused on so-called anti-icing surfaces. The widely accepted engineering approach is physical and chemical de-icing of surfaces (numerous recent developments are reviewed in [45]). The so-called ice-phobicity might be one of the possible strategies to reduce the formation of ice. In other words, the liquid should be removed before it freezes to the surface. The superhydrophobicity of the surface might well contribute to such an effect, which has been previously suggested in the literature on bioinspired passive anti-icing and ice-phobic surfaces [36,45]. The present study on *D. antarctica* provides interesting data about surface adaptations in the plant adapted to low temperatures of Antarctica. Two layers of particulate wax observed here may potentially lead to an increase of the freezing time due to the shift of ice nucleation from the cuticle surface to the tips of wax projections. This, in turn, helps to keep an air layer between the wax particles under conditions of reduced water vapor. Moreover, superhydrophobic surfaces in combination with strong air flow can lead to newly formed ice particles being blown off, since the real contact area and, consequently, the adhesion/friction on the nanoscale rough surface is rather low [46–47].

## Conclusion

A cryo-SEM examination of the vegetative (leaf blade and ligule) and generative (pedicel and outer glume) organs in *D. antarctica* revealed a prominent epicuticular wax coverage on surfaces of both vegetative organs studied. Whereas the ligule is loosely covered with separate scale-like projections or clusters of them, both leaf sides bear a hierarchical, two-layered wax coverage composed of polygonal rodlets formed by fused irregular platelets in the lower (inner) wax layer and membraneous platelets in the upper (outer) wax layer. Both leaf surfaces show a rather similar microstructure of the wax coverage and differ mainly in the thickness ratio between lower and upper wax layer. The presence of the prominent epicuticular wax on the abaxial leaf side and ligule has been found for the first time here. Also, the hierarchical structure of the wax coverage on both leaf surfaces resembling that described for the slippery (waxy) zone of several species of carnivorous plants from the genus *Nepenthes* has been discovered in *D. antarctica* in this study. The present study on *D. antarctica* provides not only interesting data about surface adaptations in the plant adapted to low temperatures of Antarctica, but also about possible strategies of anti-icing coatings of technical surfaces. Future detailed experimental studies on *D. antarctica* can provide more reliable information on the functional mechanisms behind the specific wax coverage in this plant.
